# Impedance Control for Robotic Rehabilitation: A Robust Markovian Approach

**DOI:** 10.3389/fnbot.2017.00043

**Published:** 2017-08-24

**Authors:** Andres L. Jutinico, Jonathan C. Jaimes, Felix M. Escalante, Juan C. Perez-Ibarra, Marco H. Terra, Adriano A. G. Siqueira

**Affiliations:** ^1^Robotic Rehabilitation Laboratory, Department of Mechanical Engineering, Center for Robotics of São Carlos, University of São Paulo São Paulo, Brazil; ^2^Intelligent Systems Laboratory, Department of Electrical Engineering, Center for Robotics of São Carlos, University of São Paulo São Paulo, Brazil

**Keywords:** robotic rehabilitation, impedance control, *H*_∞_, Markovian jump linear systems, series elastic actuators, robust control, force control

## Abstract

The human-robot interaction has played an important role in rehabilitation robotics and impedance control has been used in the regulation of interaction forces between the robot actuator and human limbs. Series elastic actuators (SEAs) have been an efficient solution in the design of this kind of robotic application. Standard implementations of impedance control with SEAs require an internal force control loop for guaranteeing the desired impedance output. However, nonlinearities and uncertainties hamper such a guarantee of an accurate force level in this human-robot interaction. This paper addresses the dependence of the impedance control performance on the force control and proposes a control approach that improves the force control robustness. A unified model of the human-robot system that considers the ankle impedance by a second-order dynamics subject to uncertainties in the stiffness, damping, and inertia parameters has been developed. Fixed, resistive, and passive operation modes of the robotics system were defined, where transition probabilities among the modes were modeled through a Markov chain. A robust regulator for Markovian jump linear systems was used in the design of the force control. Experimental results show the approach improves the impedance control performance. For comparison purposes, a standard $H_{\infty }$ force controller based on the fixed operation mode has also been designed. The Markovian control approach outperformed the $H_{\infty }$ control when all operation modes were taken into account.

## 1. Introduction

Physical therapy represents a well-accepted procedure for improvements in the recovery of human motor function and promotion of higher performance in Activities of Daily Life (ADLs) (Krebs et al., 2008). Mainly when people have been affected by injuries such as stroke (Hatano, 1976) and Multiple Sclerosis (MS) (Cattaneo et al., 2002). Robotic-assisted therapy is a promising field for the development of rehabilitation tasks. Among the advantages offered by robotic devices are uniformity in the repetition of long-time routines, reliable records of measured variables, and motivation for the patient's participation using interactive environments like serious games (Lum et al., 2002; Chang and Kim, 2013; Gonçalves et al., 2014). However, the fact that these robots interact with humans during therapeutic movement, they require a high degree of security and reliability. Therefore, rehabilitation robots should identify activities performed by the patient to reach only pre-defined training objectives, whose principle is known as *Assist-as-Needed Paradigm* (Radomski and Trombly, 2013).

Impedance control has been used in the implementation of this kind of paradigm in rehabilitation robotic systems. It was initially proposed for manipulator robots to obtain a safe physical interaction with the environment (Hogan, 1985). Such a controller aims at establishing a dynamic relationship between the force and velocity of an actuator. Series elastic actuators (SEA) provide a simple and efficient solution for the implementation of impedance controllers (Calanca et al., 2016). However, the impedance control of SEAs requires an explicit force control loop whose performance is sensitive to uncertainties and time-varying human dynamics.

Colgate and Hogan (1988, 1989) addressed the problem of interaction control and analyzed the energy exchange between a robotic system and its environment, defined as passive. They also determined stability criteria for a coupled system. Some years later this analysis was brought into the context of human-robot interaction considering three new issues: (1) Human dynamics is now the environment; (2) This dynamics can exhibit passive and active behaviors; and, (3) The stability criterion has been reformulated as complementary stability (Buerger and Hogan, 2007). Such new concepts emphasize the way the human dynamics is taken into account. For example, Vallery et al. (2008) analyzed limits of coupled stability and performance of an SEA actuator for rehabilitation applications considering the human dynamics a spring and damping system. Kong et al. (2009) analyzed those limits considering the human dynamics only as a mass (inertia). In Oh and Kong (2017), the human dynamics was considered as stiffness, damping, and inertia. Li et al. (2017) proposed an adaptive control scheme for SEA-driven robots which consider two operation modes in the adaptation process, namely robot-in-charge and human-in-charge. Similar approaches were proposed in Yu et al. (2015) and Pan et al. (2017), however, the authors did not take into account the fact the human-robot system can be modeled by different operation modes related to abrupt changes in the dynamic behavior.

This paper reports on the implementation of an impedance controller in a robotic platform for ankle rehabilitation based on a Markovian approach. The platform uses an SEA and enables plantarflexion and dorsiflexion movements. The following three operation modes that may occur in the robot-human interaction were defined: (1) fixed mode, in which the platform is mechanically fixed; (2) resistive mode, in which the user makes efforts against the platform movement; and (3) passive mode, in which the user does not make any effort against the platform movement. Such operation modes are modeled as states of a Markov chain. Based on this modeling, a recursive robust regulator for discrete Markov jump linear systems (RR-DMJLS) proposed in Cerri and Terra (2017) is designed to regulate the force control. It guarantees mean square stability and optimal performance for this class of stochastic system (Jutinico et al., 2017). In order to check the effectiveness of the approach, we performed a comparative study with a standard $H_{\infty }$ force controller proposed in Pérez-Ibarra et al. (2017) which is designed based only on the higher-impedance operation mode (fixed). Although this control approach provides robust stability for the whole system, including all operation modes, its performance was outperformed by RR-DMJLS. We present actual results based on force- and impedance-control for both controllers.

The paper is organized as follows: Section 2 introduces the SEA-based robotic platform and its respective dynamic model; Section 3 describes the design of the robust controllers; Section 4 reports experimental results of a comparative study between the controllers; finally, Section 5 provides the conclusions and some final remarks for future work.

## 2. System description and modeling

**Figure 1:**
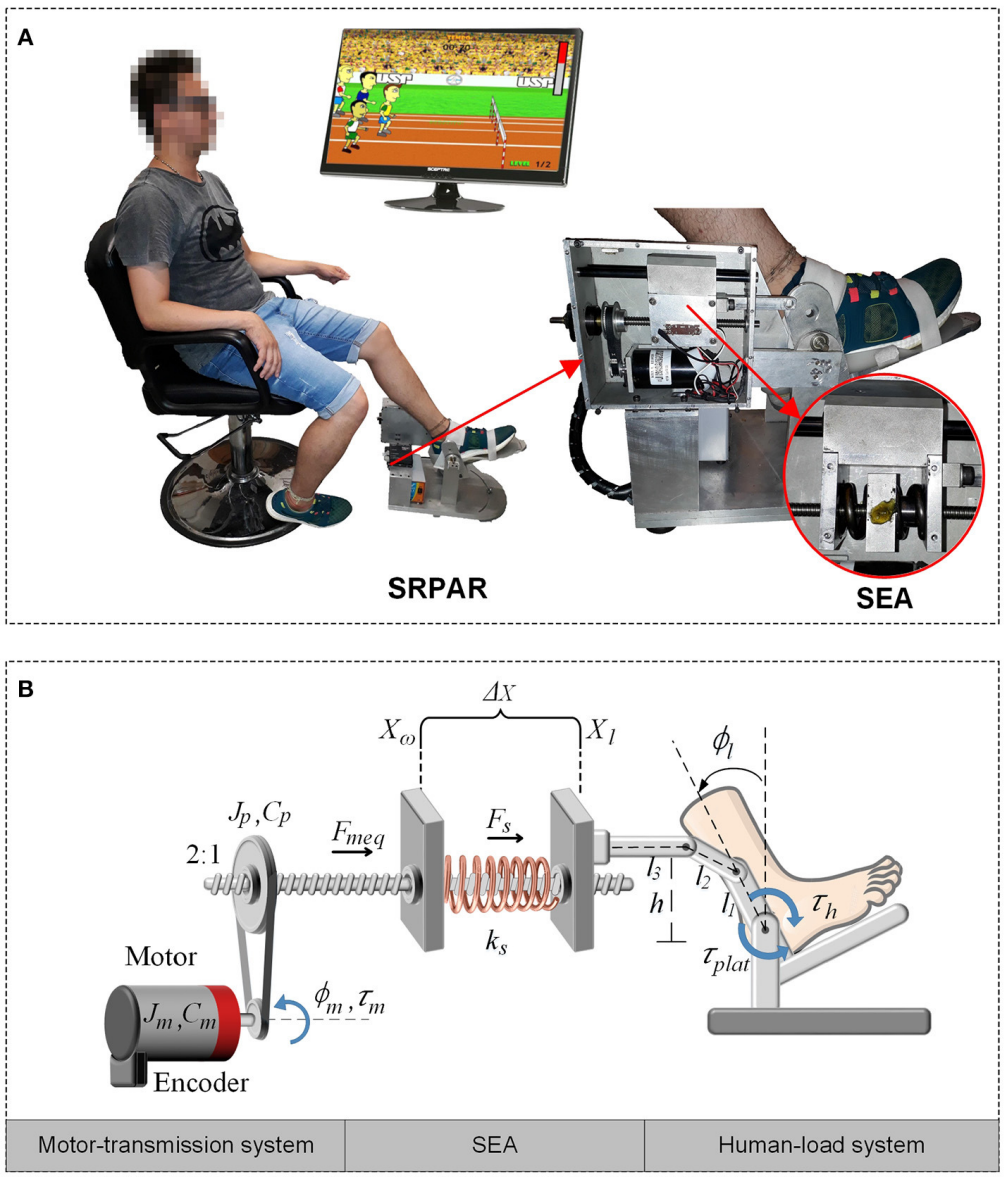
SEA-based Robotic Platform for Ankle Rehabilitation (SRPAR). **(A)** Platform (Gonçalves et al., 2014). **(B)** Squematic model.

The SEA-based Robotic Platform for Ankle Rehabilitation (SRPAR), Figure 1, is a device for robot-assisted training. It works under two conditions: guidance of a physiotherapist during pre-established dorsi/plantarflexion movements of the ankle and active participation of the patient by using serious games. Both conditions benefit individuals who have suffered a stroke. Also, the platform is a tool to evaluate the ankle force and range of movement (Gonçalves et al., 2013).

The platform uses a DC motor (Maxon Motor RE 40, graphite brushes, 150 Watt DC motor) linked to a ballscrew through a belt and pulleys with 2:1 reduction ratio. A recirculating ballscrew nut converts the rotational motion of the screw in a linear motion. A pair of steel springs is attached to the nut and to a movable piece. When the motor is driven, the nut moves forward or backward compressing the springs. The movable part is connected to a kinematic chain which converts linear force into torque which is transferred to the user. We estimate the nut's position by measuring the motor rotation with a magneto-resistant incremental encoder. Finally, we estimate the spring force by measuring the spring deformation. A logarithmic sliding potentiometer is attached to the movable piece located between the springs. The potentiometer's cursor moves along with the piece generating a voltage proportional to the spring deformation.

### 2.1. Transfer function model

In order to describe the dynamic behavior of the system, we define three sub-systems in the SRPAR: motor-transmission system, series elastic element, and human-load system (Figure 1B). A set of assumptions is made to simplify the dynamic modeling, resulting in a linear model for the SRPAR. Although these simplifications can result in a limited model, mainly due to the presence of nonlinearities, the use of robust controllers to deal with uncertain parameters can improve the performance of the system.

Concerning the motor-transmission sub-system, although some studies deal with only the effect of the inertia in the motor model (Vallery et al., 2008; Calanca et al., 2014), in this paper we also consider the influence of the motor damping. The effects of the inertia and damping parameters of the pulley and ballscrew are also considered as in Yu et al. (2013) and dos Santos et al. (2015). In addition, the nonlinear effects of friction, backslash, and efficiency losses in the motor-transmission system are minimized by controlling the motor velocity instead of directly controlling the motor torque or current, as discussed in Wyeth (2006).

Regarding the series elastic configuration, in Petit et al. (2015) is presented a generic model for robotic systems with variable stiffness. They describe the output torque of the actuator by a nonlinear function that depends on the joint deflection and mechanical stiffness variation of the springs. In this paper, the springs are modeled as constant stiffness and operate in a linear region. Since the human limb is attached to the four-bar mechanism of the SRPAR, rotation of the ankle is transformed into a linear movement in the same direction of the spring force by a nonlinear Jacobian. However, due to a small range of allowed movements related to mechanical constraints of the SRPAR, this transformation is approximated by a linear relationship (see Equation 6). The gravity effects on the human foot are also neglected since the distance between the foots center of gravity and the rotation axis of the platform is small.

Different approaches have been proposed to model the dynamics of the human-load sub-system. For example, Tagliamonte and Accoto (2014) used a set of second-order plants to represent human dynamics in order to implement an impedance controller where passivity concepts are explored. In Lee et al. (2016), similar second-order models are used to measure the ankle mechanical impedance in a unified way. In this paper, we model the human dynamics as a set of second-order linear plants subject to parameter uncertainties.

In the following, we present a transfer function between the commanded motor velocity $\omega_{m}^{d}$ and the spring force *F*_*s*_.

#### 2.1.1. Dynamics of the motor-transmission system

Motor-transmission can be modeled as a second-order control system,

(1) $$M_{meq}{\ddot{X}}_{w}+B_{meq}{\dot{X}}_{w}=F_{meq}-F_{s},$$

where *X*_*w*_ denotes the displacement of ball screw nut, *M*_*meq*_ and *B*_*meq*_ are respectively the equivalent inertia and damping of the system as defined in Equation (2), and *F*_*meq*_ is the output force as defined in Equation (3),

(2) $$M_{meq}=M_{t}+\rho^{2}(J_{p}+N_{p}^{2}J_{m})\text{ and }B_{meq}=B_{t}+\rho^{2}(C_{p}+N_{p}^{2}C_{m}).$$

(3) $$F_{meq}=\rho N_{p}K_{t}i_{m}=\rho N_{p}K_{t}\left(\left(K_{p}+\frac{K_{i}}{s})(\omega_{m}^{d}-\omega_{m}\right)\right).$$

In Equations (2) and (3), *M*_*t*_ and *B*_*t*_ are the mass and damping of the ball screw nut, *J* and *C* are torsional inertia and damping where subscripts _*m*_ and _*p*_ stand for motor and pulley, respectively. *N*_*p*_ is the pulley ratio, $\rho =\frac{2\pi }{l}$ is a rotational-to-linear factor where *l* is the ball screw lead. *K*_*t*_ is the motor constant, *i*_*m*_ is the motor current determined by the inner velocity control of the motor, and *K*_*p*_ and *K*_*i*_ are the proportional and integral gains of the controller; ω_*m*_ and $\omega_{m}^{d}$ are actual and desired motor velocities, respectively.

#### 2.1.2. Dynamics of the human-load system

Dynamics of the human ankle is modeled by a second-order system with inertia, damping and stiffness parameters given by:

(4) $$J_{l}{\ddot{\phi }}_{l}+C_{l}{\dot{\phi }}_{l}+K_{h}\phi_{l}+G_{l}(\phi_{l})=\tau_{h}-\tau_{plat},$$

where ϕ_*l*_ denotes the angular position of the ankle, τ is the torque, _*plat*_ and _*h*_ stand for platform and human being, and *G*_*l*_(ϕ_*l*_) represents the gravitational effects. *J*_*l*_ = *J*_*plat*_ + *J*_*h*_ and *C*_*l*_ = *C*_*plat*_ + *C*_*h*_ are the equivalent inertia and damping of the sub-system, respectively, and *K*_*h*_ is the ankle stiffness.

The linear displacement of the load, *X*_*l*_, is expressed in terms of the angular movement of the human ankle joint ϕ_*l*_, by:

(5) $$X_{l}=l_{1}\text{sin }\phi_{l}+l_{2}\sqrt{1-{\left(\frac{h-l_{1}\text{cos }\phi_{l}}{l_{2}}\right)}^{2}}+l_{3},$$

where *l*_1_, *l*_2_, *l*_3_, and *h* are known distances of the platform (see Figure 1B). Based on linear and angular load velocities, we compute the following Jacobian:

(6) $$\begin{array}{l} J(\phi_{l})=\frac{{\dot{X}}_{l}}{w_{l}}=l_{1}\text{cos }\phi_{l}-\frac{\left(h-l_{1}\text{cos }\phi_{l}\right)\left(l_{1}\text{sin }\phi_{l}\right)}{\sqrt{l_{2}^{2}-{\left(h-l_{1}\text{cos }\phi_{l}\right)}^{2}}},\\ \text{ where }w_{l}={\dot{\phi }}_{l}. \end{array}$$

Since we are considering a small range of allowed movements, approximately |ϕ_*l*_| < 0.35 rad, this equation can be simplified by $J\left(\phi_{l}\right)\approx l_{1}$. As aforementioned the distance between the foot's center of gravity and the rotation axis of the platform is small, in this case we can neglect the gravitational effect *G*_*l*_(ϕ_*l*_). Hence, from Equations (4) and (6), the dynamics of the human-load system is given by:

(7) $$M_{l}{\ddot{X}}_{l}+B_{l}{\dot{X}}_{l}+K_{l}X_{l}=F_{s}-J^{-1}\tau_{h},$$

where *M*_*l*_, *B*_*l*_, and *K*_*l*_ are respectively equivalent inertia, damping and stiffness of the human-load system, defined by:

(8) $$M_{l}=J^{-2}J_{l},B_{l}=J^{-2}J_{l}\dot{J}+J^{-2}C_{l}\text{ and }K_{l}=J^{-2}K_{h}.$$

#### 2.1.3. Dynamics of the series elastic actuator

Taking the Laplace transform of Equations (1) and (7), and solving for *X*_*w*_ and *X*_*l*_, we obtain:

(9) $$X_{w}(s)=\frac{F_{meq}-F_{s}}{M_{meq}s^{2}+B_{meq}s},\text{ and }X_{l}(s)=\frac{F_{s}-J^{-1}\tau_{h}}{M_{l}s^{2}+B_{l}s+K_{l}}.$$

We make use of the Hooke's Law to define output spring force *F*_*s*_:

(10) $$F_{s}=K_{s}\Delta X=K_{s}\left(X_{w}-X_{l}\right).$$

From Equations (9) and (10), we have:

(11) $$F_{s}(s)=K_{s}\frac{F_{meq}Z_{l}+J^{-1}\tau_{h}Z_{meq}}{Z_{l}Z_{meq}\;s+K_{s}(Z_{l}+Z_{meq})},$$

where *Z*_*meq*_ = *M*_*meq*_*s* + *B*_*meq*_ and $Z_{l}=M_{l}s+B_{l}+\frac{Kl}{s}$ are the mechanical impedances of the motor-transmission and human-load system, respectively. Finally, the spring force *F*_*s*_(*s*) is expressed as a function of the desired motor velocity $\omega_{m}^{d}$ and human torque τ_*h*_, as:

(12) $$F_{s}(s)=K_{s}\frac{\left(\rho N_{p}KtZ_{l}\right)\omega_{m}^{d}+\left(J^{-1}Z_{meq}\right)\tau_{h}}{Z_{l}Z_{meq}\;s+K_{s}\left(\frac{Z_{l}s}{\left(K_{p}s+K_{i}\right)}+Z_{meq}\right)},$$

thus, the system dynamics is defined by the transfer functions *G*_*n*_(s) and *G*_*h*_(*s*), given by:

(13) $$G_{n}(s)=\frac{F_{s}}{\omega_{m}^{d}}=\frac{\rho N_{p}K_{PI}K_{s}K_{t}Z_{l}}{K_{PI}Z_{l}Z_{meq}s+K_{s}\left(Z_{l}+Z_{meq}K_{PI}\right)},$$

(14) $$G_{h}(s)=\frac{F_{s}}{\tau_{h}}=\frac{K_{s}J^{-1}Z_{meq}}{K_{PI}Z_{l}Z_{meq}s+K_{s}\left(Z_{l}+Z_{meq}K_{PI}\right)},$$

where $K_{PI}=K_{p}+\frac{K_{i}}{s}$. The nominal SEA model is obtained fixing the output load making *X*_*l*_ = 0 (Robinson et al., 1999; dos Santos et al., 2015). Thus making *Z*_*l*_ → ∞ in Equation (13), we obtain a transfer function *G*(*s*) that only considers platform parameters:

(15) $$G(s)={G_{n}(s)|}_{Z_{l}\to \infty }=\frac{\rho N_{p}K_{s}K_{t}K_{PI}}{K_{PI}M_{meq}s^{2}+K_{PI}B_{meq}s+K_{s}}.$$

### 2.2. State space model

Consider again the system shown in Figure 1B. In order to simplify the model, the inner velocity loop control allows us to model the motor as a pure velocity source; therefore the torque of the motor τ_*m*_ is given by:

(16) $$\tau_{m}=K_{t}i_{m}=J_{m}\frac{dw_{m}}{dt}+C_{m}w_{m}\approx C_{m}w_{m}$$

and, in consequence,

(17) $$F_{meq}=\rho N_{p}K_{t}i_{m}=\rho N_{p}C_{m}w_{m}.$$

From Equations (1) and (17), and taking into account that the angular position of the motor ϕ_*m*_ is described in function of the displacement *X*_*w*_ by ϕ_*m*_ = ρ*N*_*p*_*X*_*w*_, we obtain:

(18) $${\ddot{\phi }}_{m}=\left(\frac{\rho^{2}N_{p}^{2}C_{m}}{M_{meq}}-\frac{B_{meq}}{M_{meq}}\right)w_{m}-\frac{\rho N_{p}}{M_{meq}}F_{s},\text{ where }w_{m}={\dot{\phi }}_{m}.$$

From Equations (7) and (10), we obtain the following expression:

(19) $$\begin{array}{l} {\ddot{F}}_{s}=\frac{K_{s}}{\rho N_{p}}{\ddot{\phi }}_{m}-\frac{B_{l}}{M_{l}}{\dot{F}}_{s}-\left(\frac{K_{l}}{M_{l}}+\frac{K_{s}}{M_{l}}\right)F_{s}+\frac{K_{s}B_{l}}{\rho N_{p}M_{l}}w_{m}\\ \;+\;\frac{K_{l}K_{s}}{\rho N_{p}M_{l}}\phi_{m}+\frac{K_{s}J^{-1}}{M_{l}}\tau_{h}. \end{array}$$

Using Equations (6), (8), (18), and (19) it is possible to define the following state space representation of the SRPAR-human system:

(20) $$\underset{{\dot{x}}_{a}}{\underset{︸}{\left[\begin{array}{c} {\ddot{F}}_{s}\\ {\dot{F}}_{s}\\ {\dot{\phi }}_{m}\\ {\dot{\phi }}_{l} \end{array}\right]}}=\underset{F_{a}}{\underset{︸}{\left[\begin{array}{cccc} -\frac{C_{l}}{J_{l}} & \left(\frac{-K_{s}}{M_{meq}}-\frac{K_{h}+K_{s}J^{2}}{J_{l}}\right) & \frac{K_{h}K_{s}}{\rho N_{p}J_{l}} & 0\\ 1 & 0 & 0 & 0\\ 0 & 0 & 0 & 0\\ -{\left(K_{s}J\right)}^{-1} & 0 & 0 & 0 \end{array}\right]}}\underset{x_{a}}{\underset{︸}{\left[\begin{array}{c} {\dot{F}}_{s}\\ F_{s}\\ \phi_{m}\\ \phi_{l} \end{array}\right]}}+\underset{B_{a}}{\underset{︸}{\left[\begin{array}{c} \frac{K_{s}}{\rho N_{p}}\left(\frac{C_{l}}{J_{l}}-\frac{B_{meq}}{M_{meq}}\right)+\frac{\rho N_{p}K_{s}C_{m}}{M_{meq}}\\ 0\\ 1\\ {\left(\rho N_{p}J\right)}^{-1} \end{array}\right]}}w_{m}+\underset{G_{a}}{\underset{︸}{\left[\begin{array}{c} \frac{K_{s}J}{J_{l}}\\ 0\\ 0\\ 0 \end{array}\right]}}\tau_{h},$$

(21) $$\begin{array}{l} \underset{y}{\underset{︸}{\left[\begin{array}{l} w_{l}\\ \phi_{l} \end{array}\right]}}=\underset{C}{\underset{︸}{\left[\begin{array}{cccc} -{\left(K_{s}J\right)}^{-1} & 0 & 0 & 0\\ 0 & 0 & 0 & 1 \end{array}\right]}}\begin{array}{l} \left[\begin{array}{c} {\dot{F}}_{s}\\ F_{s}\\ \phi_{m}\\ \phi_{l} \end{array}\right] \end{array}+\begin{array}{l} \underset{D}{\underset{︸}{\left[\begin{array}{c} {\left(\rho N_{p}J\right)}^{-1}\\ 0 \end{array}\right]}}w_{m}, \end{array} \end{array}$$

where *F*_*s*_ is the spring force and, ϕ_*m*_ and ϕ_*l*_ are angular positions for motor and load, respectively. The system control input is the motor angular velocity *w*_*m*_ and τ_*h*_ is the human torque which is considered as an input disturbance.

### 2.3. Experimental validation

**Figure 2:**
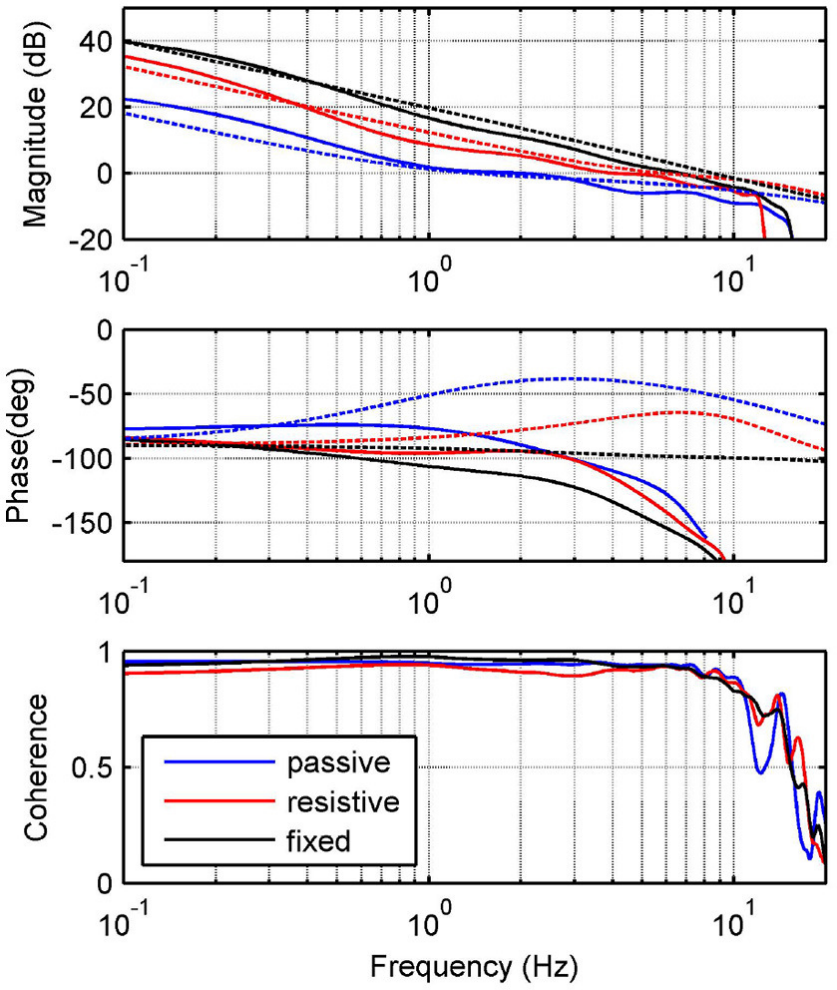
Experimental identification. Frequency response measurements of theoretical (dashed) and experimental (solid) transfer functions between input $\omega_{m}^{d}$ and output *F*_*s*_. Graphs show responses for passive (blue), resistive (red) and fixed (black) modes.

In order to validate the proposed theoretical model, we identify the frequency response function (FRF) of the force modeled in Equation (13). We consider in the human-load interaction, three specific operation modes: (1) a fixed mode, in which the platform is fixed in a neutral position, i.e., ϕ_*l*_ ≈ 0 and *Z*_*l*_ → ∞; (2) a resistive mode, where the human being makes effort against the platform in order to hold it in the neutral position; and (3) a passive mode, when the user leaves the platform leads the movement. For all modes, we apply a desired motor velocity given by a chirp signal with an amplitude of 209.4 rad/s (2,000 rpm) and sweeping frequencies between 0 and 20 Hz (Figure 2).

Due to variations in the activation level of the muscles acting on the ankle joint, abrupt changes in ankle mechanical impedance are expected. In addition, operation modes defined are properly matched with the phases of human gait pattern. Thus, the fixed mode can be associated with the mid-stance phase; the resistive mode with the initial contact, terminal stance and double support of the stance phase; and the passive mode with the swing phase. Table 1 shows the platform specifications, as well as the human parameters used for each mode and their corresponding lower limits. Parameters for Mode 1 were chosen to satisfy *Z*_*l*_ → ∞, in order to obtain similar results to those presented in Robinson et al. (1999). For Modes 2 and 3, they were chosen from Lee et al. (2016) taking into account the actual human impedance during stance and swing phases of walking, respectively.

Table 1Platform and human parameters.

**Table:** 

**Parameter**	**Value**	**Parameter**	**Value**
*N*_*p*_	2	*J*_*p*_	0.000055 kg·m^2^
*l*	0.0025 *m*	*C*_*m*_	0.00287 N · m · s/rad
J	0.03 m/rad	*J*_*m*_	0.0000138 kg · m^2^
*J*_*plat*_	0.0013 kg· m^2^	*K*_*s*_	320000 N/m
*C*_*plat*_	3.5 N·m·s/rad	*M*_*t*_, *B*_*t*_, *C*_*p*_	≈ 0
*K*_*p*_	30.42 A·s/rad	*K*_*i*_	1.23 A· s/rad
*K*_*t*_	0.0302 N·m/A	*T*_*s*_	0.001 s

**Table:** 

**Human parameter**	**Mode 1**	**Mode 2**	**Mode 3**	**Lower limits**
*J*_*h*_ (kg· m^2^)	50	0.08	0.02	0.0001
*C*_*h*_ (N· m· s/rad)	1e5	5	0.5	0.001
*K*_*h*_ (N· m/rad)	4e5	200	20	0

## 3. Control approaches

**Figure 3:**
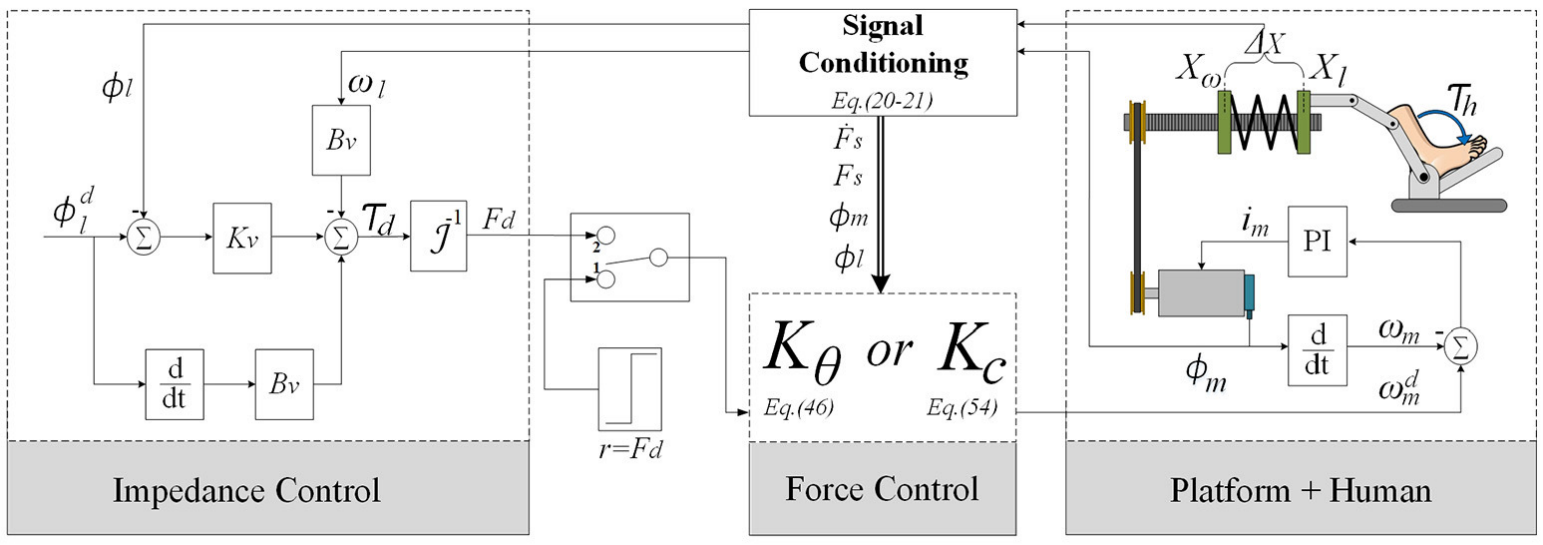
Overall control scheme for the SRPAR.

The block diagram of the control system for the SRPAR is presented in Figure 3. The platform uses an EPOS driver that performs two internal control loops for motor velocity and current. They are based on PI controllers with Kp and Ki gains shown in (see Table 1). Platform sensors provide data to a signal conditioning block where SRPAR-human system variables are computed. The sampling frequency used in this process is 1 kHz.

In order to ensure an appropriate interaction between human and platform, we implement a standard impedance control configuration for SEAs that uses the following internal force control law:

(22) $$F_{d}=J^{-1}\left(K_{v}(\phi_{l}^{d}-\phi_{l})+B_{v}(\omega_{l}^{d}-\omega_{l})\right),$$

where *F*_*d*_ is the desired force computed from a sequence of desired trajectories for load angular position $\phi_{l}^{d}$ and velocity $\omega_{l}^{d}$. It is determined by the virtual stiffness *K*_*v*_ and damping *B*_*v*_.

We aim to improve the performance and to guarantee the stability of the system for different modes of human activities, where changes among them are modeled as random jumps. In this sense, we design a recursive robust regulator for discrete-time Markov jump linear systems (RR-DMJLS). Disturbances and uncertainties due to human-robot interactions, mainly because of human parameters (inertia, stiffness, and damping), are considered in this control approach. For comparison purposes, we also design an $H_{\infty }$ force controller that does not take into account these different modes of human activities. We synthesize a fixed-gain controller using a similar approach presented in Mehling and O'Malley (2014) and dos Santos et al. (2015). Both controllers are presented in the following.

### 3.1. Recursive RR-DMJLS force control design

**Figure 4:**
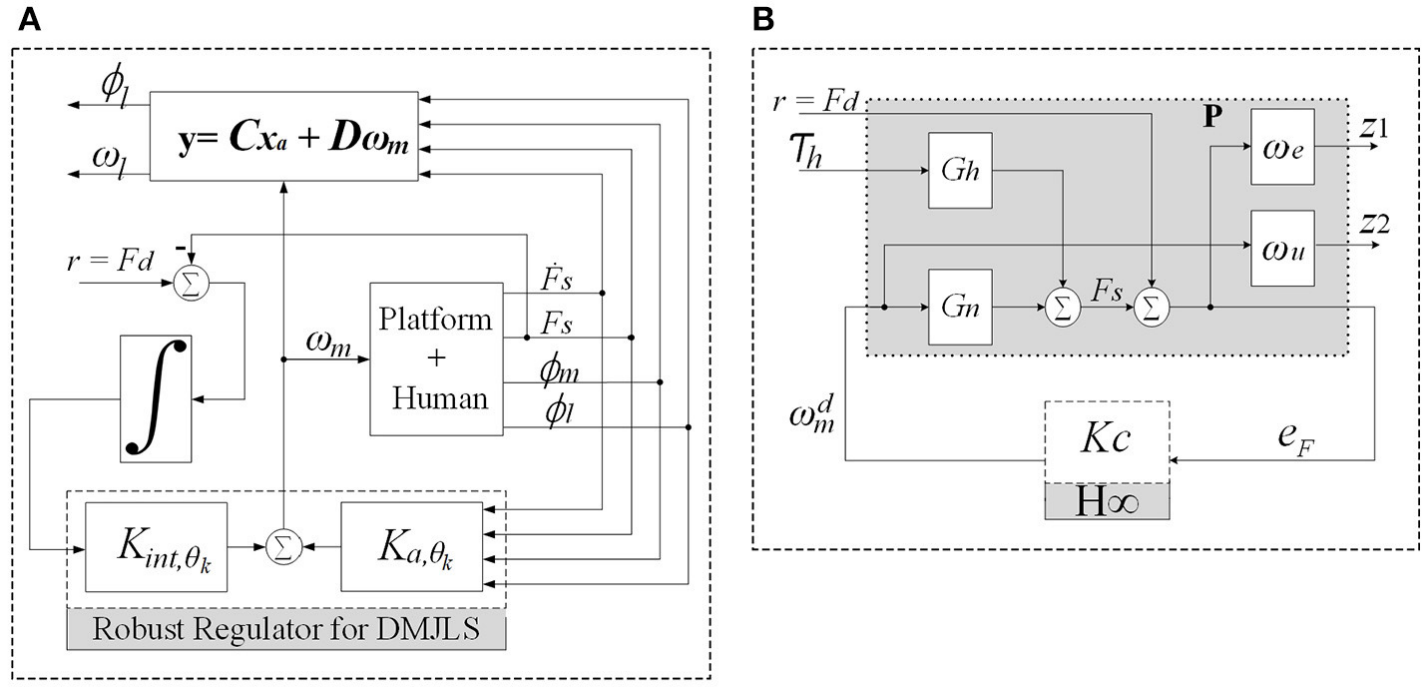
Force control approaches. **(A)** Recursive Robust Regulator for DMJLS: the block “Platform + Human” corresponds to the state variable model in Equation 20, *K*_*int*,_θ_*k*___ and *K*_*a*,_θ_*k*___ are the control gains. **(B)** $H_{\infty }$ control configuration of the system: *G*_*n*_ is the process plant, P is the augmented plant including the weight functions and *K*_*c*_ is the controller.

In this section, we design a robust force controller for the SRPAR (Figure 4A). We present first nominal and uncertain representations for different operation modes of the system. Then, we model the system as a discrete-time Markovian jump linear system (DMJLS). In order to guarantee stability and robust performance, we use the recursive RR-DMJLS algorithm developed in Cerri and Terra (2017).

#### 3.1.1. Nominal model

Consider the model presented in Equation (20),

(23) $${\dot{x}}_{a}=F_{a_{\theta }}x_{a}+B_{a_{\theta }}w_{m}+G_{a_{\theta }}\tau_{h},$$

for each operation mode θ ∈ Θ: = {1, …, *s*}, where *s* is the number of nominal models, $F_{a_{\theta }}\in {\mathbb{R}}^{nxn}$, $B_{a_{\theta }}\in {\mathbb{R}}^{nxm}$, and $G_{a_{\theta }}\in {\mathbb{R}}^{nxm}$ are nominal parameter matrices, $x_{a}\in {\mathbb{R}}^{n}$ is the state vector, $w_{m}\in {\mathbb{R}}^{m}$ is the input control and $\tau_{h}\in {\mathbb{R}}^{m}$ is the input disturbance.

We discretized this model by using *F*_*a*_θ, *k*__ = *I* + *F*_*a*_θ__*T*_*s*_Ω, *B*_*a*_θ, *k*__ = Ω*T*_*s*_*B*_*a*_θ__, and *G*_*a*_θ, *k*__ = Ω*T*_*s*_*G*_*a*_θ__, with $\Omega =\overset{9}{\underset{k_{n}=0}{\sum }}\frac{F_{a_{\theta }}^{k_{n}}T_{s}^{k_{n}}}{\left(k_{n}+1\right)!}$, where *T*_*s*_ = 1 ms is the sample time, for each *k* ∈ ℤ^+^. To eliminate the steady state error, we augment the model by including an integral action:

(24) $$\begin{array}{l} \underset{x_{k+1}}{\underset{︸}{\left[\begin{array}{c} x_{a_{k+1}}\\ x_{int_{k+1}} \end{array}\right]}}=\underset{F_{\theta ,k}}{\underset{︸}{\left[\begin{array}{cc} F_{a_{\theta ,k}} & 0\\ C_{a}T_{s} & 1 \end{array}\right]}}\underset{x_{k}}{\underset{︸}{\left[\begin{array}{c} x_{a_{k}}\\ x_{int_{k}} \end{array}\right]}}+\underset{B_{\theta ,k}}{\underset{︸}{\left[\begin{array}{c} B_{a_{\theta ,k}}\\ 0 \end{array}\right]}}\underset{u_{k}}{\underset{︸}{w_{m_{k}}}}\\ \;+\underset{Br_{\theta ,k}}{\underset{︸}{\left[\begin{array}{c} 0\\ T_{s} \end{array}\right]}}r_{k}+\underset{G_{\theta ,k}}{\underset{︸}{\left[\begin{array}{c} G_{a_{\theta ,k}}\\ 0 \end{array}\right]}}\tau_{h_{k}}, \end{array}$$

where *r*_*k*_ is a force reference signal and *C*_*a*_ = [0 − 1 0 0]. *F*_θ, *k*_ and *B*_θ, *k*_ are nominal matrices for three nominal models according to Equation (20), which are based on parameters presented in Table 1:

(25) $$\begin{array}{l} \text{Mode 1: }F_{1,k}=\left[\begin{array}{ccccc} 0.134 & -3.81 & 219 & 0 & 0\\ 4\cdot 10^{-4} & 0.998 & 0.144 & 0 & 0\\ 0 & 0 & 1 & 0 & 0\\ 0 & 0 & \;-1\cdot 10^{-5} & 1 & 0\\ 0 & -0.001 & 0 & 0 & 1 \end{array}\right],\\ \;B_{1,k}=\left[\begin{array}{c} 55.11\\ 0.036\\ 0.001\\ 3\cdot 10^{-6}\\ -1\cdot 10^{-5} \end{array}\right]; \end{array}$$

(26) $$\begin{array}{l} \text{Mode 2: }F_{2,k}=\left[\begin{array}{ccccc} 0.898 & -4.26 & 7.43 & 0 & 0\\ 9\cdot 10^{-4} & 0.998 & 0.003 & 0 & 0\\ 0 & 0 & 1 & 0 & 0\\ 9\cdot 10^{-8} & 0 & 0 & 1 & 0\\ 0 & -0.001 & 0 & 0 & 1 \end{array}\right],\\ \;B_{2,k}=\left[\begin{array}{c} 6.31\\ 0.003\\ 0.001\\ 6\cdot 10^{-6}\\ -1\cdot 10^{-6} \end{array}\right]; \end{array}$$

(27) $$\begin{array}{l} \text{Mode 3: }F_{3,k}=\left[\begin{array}{ccccc} 0.822 & -13.1 & 2.72 & 0 & 0\\ 9\cdot 10^{-3} & 0.993 & \;0.001 & 0 & 0\\ 0 & 0 & 1 & 0 & 0\\ 9\cdot 10^{-8} & 0 & 0 & 1 & 0\\ 0 & -0.001 & 0 & 0 & 1 \end{array}\right],\\ \;B_{3,k}=\left[\begin{array}{c} 10.87\\ 0.005\\ 0.001\\ 6\cdot 10^{-6}\\ -2\cdot 10^{-6} \end{array}\right]. \end{array}$$

#### 3.1.2. Uncertain model

Consider the system presented in Section 3.1.1 subject to parametric uncertainties given by:

(28) $$\begin{array}{l} \left[\begin{array}{c} x_{a_{k+1}}\\ x_{int_{k+1}} \end{array}\right]=\underset{F_{\theta ,k}^{\delta }=F_{\theta ,k}+\delta F_{\theta ,k}}{\underset{︸}{\left[\begin{array}{cc} F_{a_{\theta ,k}}+\delta F_{11_{\theta ,k}} & \delta F_{12_{\theta ,k}}\\ C_{a}T_{s}+\delta F_{21_{\theta ,k}} & 1+\delta F_{22_{\theta ,k}} \end{array}\right]}}\left[\begin{array}{c} x_{a_{k}}\\ x_{int_{k}} \end{array}\right]\\ \;+\underset{B_{\theta ,k}^{\delta }=B_{\theta ,k}+\delta B_{\theta ,k}}{\underset{︸}{\left[\begin{array}{c} B_{a_{\theta ,k}}+\delta B_{11_{\theta ,k}}\\ \delta B_{21_{\theta ,k}} \end{array}\right]}}u_{k}+\left[\begin{array}{c} 0\\ T_{s} \end{array}\right]r_{k}\\ \;+\underset{G_{\theta ,k}^{\delta }=G_{\theta ,k}+\delta G_{\theta ,k}}{\underset{︸}{\left[\begin{array}{c} G_{a_{\theta ,k}}+\delta G_{11_{\theta ,k}}\\ \delta G_{21_{\theta ,k}} \end{array}\right]}}\tau_{h_{k}}. \end{array}$$

**Figure 5:**
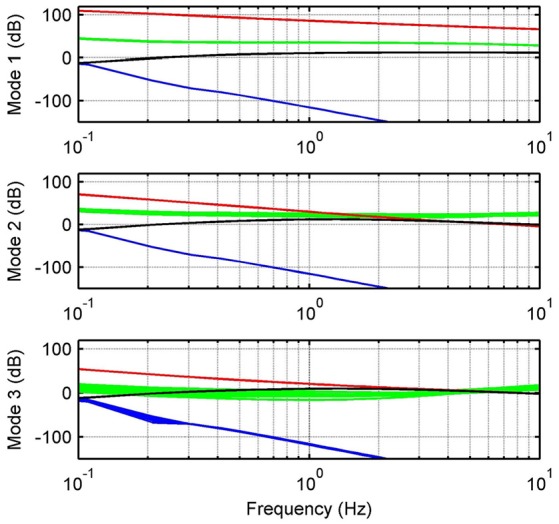
Relative errors and upper bounds. Relative errors between nominal and uncertain models with respect to inputs *u*_*k*_ (green) and *r*_*k*_ (blue). Graphs also show upper bounds for inputs *u*_*k*_ (red) and *r*_*k*_ (black).

Uncertain matrices δ*F*_θ, *k*_ and δ*B*_θ, *k*_ are modeled by:

(29) $$\left[\delta F_{\theta ,k}\;\delta B_{\theta ,k}\right]=H_{\theta ,k}\Delta_{\theta ,k}\left[E_{F_{\theta ,k}}\;E_{B_{\theta ,k}}\right],$$

where $H_{\theta ,k}\in {\mathbb{R}}^{nxk}$ is a nonzero matrix, $E_{F_{\theta ,k}}\in {\mathbb{R}}^{lxn}$ and $E_{B_{\theta ,k}}\in {\mathbb{R}}^{lxm}$ are known matrices, Δ_θ, *k*_ is an arbitrary matrix that satisfies ||Δ_θ, *k*_|| ≤ 1. In order to identify matrices *H*_θ, *k*_, *E*_*F*_θ, *k*__ and *E*_*B*_θ, *k*__, we analyze frequency responses of the nominal and uncertain models described in Equations (24) and (28), where τ_*h*_*k*__ = 0, by:

(30) $$\begin{array}{l} x_{n}(z)={\left(zI-F_{\theta ,k}\right)}^{-1}(B_{\theta ,k}u(z)+Br_{\theta ,k}r(z)),\\ \;\to G_{n_{\theta }}(e^{j\omega T_{s}})={\left(e^{j\omega T_{s}}I-F_{\theta ,k}\right)}^{-1}[B_{\theta ,k}\;B_{r_{\theta ,k}}], \end{array}$$

(31) $$\begin{array}{l} x_{un}(z)={\left(zI-F_{\theta ,k}^{\delta }\right)}^{-1}(B_{\theta ,k}^{\delta }u(z)+Br_{\theta ,k}r(z)),\\ \;\to G_{un_{\theta }}(e^{j\omega T_{s}})={\left(e^{j\omega T_{s}}I-F_{\theta ,k}^{\delta }\right)}^{-1}[B_{\theta ,k}^{\delta }\;B_{r_{\theta ,k}}], \end{array}$$

with $z=e^{j\omega T_{s}}\approx \frac{1+j\omega T_{s}/2}{1-j\omega T_{s}/2}$. For each operation mode, we compute a transfer function *W*_*a*, θ_ in order to satisfy:

(32) $$\|\sigma_{W_{a,\theta }(e^{j\omega T_{s}})}\|\geq \|\sigma_{G_{n_{\theta }}(e^{j\omega T_{s}})}-\sigma_{G_{un_{\theta }}(e^{j\omega T_{s}})}\|,\;\forall \omega$$

where σ_*G*_*n*_θ___ and σ_*G*_*un*_θ___ are singular values of the nominal and uncertain models, respectively, see Figure 5. Notice that upper bounds defined by singular values of *W*_*a*, θ_ are effective for frequencies until 1.7 Hz for resistive mode and 3.8 Hz for passive mode.

Finally, matrices *H*_θ, *k*_, *E*_*F*_θ, *k*__, and *E*_*B*_θ, *k*__ are found using a genetic algorithm considering Equation (32):

(33) $$H_{1,k}=H_{2,k}=H_{3,k}={\left[\begin{array}{ccccc} -1187 & -10.3 & -0.5 & 10^{5} & 10 \end{array}\right]}^{T},$$

and

(34) $$\{\begin{array}{ll} E_{F_{1,k}}=\left[\begin{array}{ccccc} -0.86 & -330.0 & 0 & \;0 & 1308 \end{array}\right], & E_{B_{1,k}}=\left[\begin{array}{c} -118.00 \end{array}\right],\text{ for Mode 1};\\ E_{F_{2,k}}=\left[\begin{array}{ccccc} -0.43 & -661.0 & 0 & 72 & 4448 \end{array}\right], & E_{B_{2,k}}=\left[\begin{array}{c} -\;59.30 \end{array}\right],\text{ for Mode 2};\\ E_{F_{3,k}}=\left[\begin{array}{ccccc} -0.31 & -\;79.4 & 0 & 72 & 3768 \end{array}\right], & E_{B_{3,k}}=\left[\begin{array}{c} -\;47.46 \end{array}\right],\text{ for Mode 3}. \end{array}$$

#### 3.1.3. Probability matrix

Time transitions among modes defined in Section 2.3 for the system presented in Equation (28) can be modeled by a Markov chain ${\left\{\theta_{k}\right\}}_{k=0}^{N-1}$, where θ_*k*_ is called the jump parameter and belongs to a finite set Θ: = {1, …, *s*}. Transitions among these different modes are determined by a probability matrix for state transitions $\mathbb{P}=\left[p_{ij,k}\right]\in {\mathbb{R}}^{s\times s}$ where each input must satisfies the following constraints:

(35) $$\begin{array}{l} Prob\;[\theta_{k+1}=j|\theta_{k}=i]=p_{ij,k},\;Prob\;[\theta_{0}=i]=p_{i,k},\\ \sum_{j=1}^{s}p_{ij,k}=1,\;0\leq p_{ij,k}\leq 1. \end{array}$$

ℙ is defined heuristically based on empirical observations of the respective operation modes:

(36) $$\mathbb{P}=\left[\begin{array}{ccc} 0.95 & 0.05 & 0\\ 0 & 0.9 & 0.1\\ 0 & 0.1 & 0.9 \end{array}\right].$$

Different intervals for load velocity and position can describe different behaviors of the human-robot system. Namely, if the user is passive (1), resistive (2) or if the platform is fixed (3), ω_*l*_ and ϕ_*l*_ provide the jump parameter where,

(37) $$\theta_{k}=\{\begin{array}{lll} 1 & \text{ if } & \omega_{l_{k}}^{f}\leq 1\text{ and }|\phi_{l_{k}}|<0.1,\\ 2 & \text{ else if } & \omega_{l_{k}}^{f}\leq 2\text{ and }|\phi_{l_{k}}|\geq 0.1,\\ 3 & \text{ else if } & \omega_{l_{k}}^{f}>2\text{ and }|\phi_{l_{k}}|\approx 0, \end{array}$$

for each k=0,…,N-1, where $w_{l_{k}}^{f}$ is the low-pass filtered signal from load velocity absolute value, with *f*_*c*_ = 0.1 Hz and *T*_*s*_ = 1 ms, given by:

(38) $$w_{l_{k}}^{f}=(1-\varrho )w_{l_{k-1}}^{f}+\varrho |w_{l_{k-1}}|,\text{ with }\varrho =2\pi f_{c}T_{s}.$$

Since *w*_*l*_*k*__ is related to the frequency response of the system, the jump parameter θ_*k*_ is also related to the robustness of the regulator. In fact, robust stability and optimal performance is only guaranteed in the interval of frequencies in which relative errors are represented by upper bounds ||σ_*W*_*a, i*_(*jω*)_|| (Figure 5).

#### 3.1.4. RR-DMJLS algorithm

Equation (28) is rewritten as a discrete-time Markov jump linear system subject to parametric uncertainties, by:

(39) $$\begin{array}{l} x_{k+1}=\left(F_{\theta_{k},k}+\delta F_{\theta_{k},k}\right)x_{k}+\left(B_{\theta_{k},k}+\delta B_{\theta_{k},k}\right)u_{k},\\ \text{ with }\theta_{k}\in \{1,2,3\} \end{array}$$

for all k=0,…,N-1, where *F*_θ_*k*_, *k*_ and *B*_θ_*k*_, *k*_ are nominal parameter matrices for each mode given by Equation (25), *x*_*k*_ is the state vector, *u*_*k*_ is an input control, and δ*F*_θ_*k*_, *k*_ and δ*B*_θ_*k*_, *k*_ are uncertain matrices modeled as:

(40) $$\left[\delta F_{\theta_{k},k}\;\delta B_{\theta_{k},k}\right]=H_{\theta_{k},k}\Delta_{\theta_{k},k}\left[E_{F_{\theta_{k},k}}\;E_{B_{\theta_{k},k}}\right],$$

according to parameters presented in Equations (33) and (34).

Consider the robust control problem to regulate the DMJLS subject to parametric uncertainties defined in Equation (39). The solution for this problem is achieved through the min-max optimization problem:

(41) $$\underset{x_{k+1},u_{k}}{\min}\underset{\delta F_{\theta_{k},k},\delta B_{\theta_{k},k}}{\max}\{{\tilde{J}}_{K}^{\mu }(x_{k+1},u_{k},\delta F_{\theta_{k},k},\delta B_{\theta_{k},k})\},$$

for each k=N-1,…,0 and θ_*k*_ ∈ Θ, where ${\tilde{J}}_{K}^{\mu }$ is the uncertain penalized-regularized quadratic functional:

(42) $$\begin{array}{l} {\tilde{J}}_{K}^{\mu }(x_{k+1},u_{k},\delta F_{\theta_{k},k},\delta B_{\theta_{k},k})={\left[\begin{array}{c} x_{k+1}\\ u_{k} \end{array}\right]}^{T}\left[\begin{array}{cc} \Psi_{\theta_{k},k+1} & 0\\ 0 & R_{\theta_{k},k} \end{array}\right]\\ \;\left[\begin{array}{c} x_{k+1}\\ u_{k} \end{array}\right]+(\left[\begin{array}{cc} 0 & 0\\ I & -B_{\theta_{k},k}^{\delta } \end{array}\right]\left[\begin{array}{c} x_{k+1}\\ u_{k} \end{array}\right]\\ {\;-\left[\begin{array}{c} -I\\ F_{\theta_{k},k}^{\delta } \end{array}\right]x_{k})}^{T}\left[\begin{array}{cc} Q_{\theta_{k},k} & 0\\ 0 & \mu I \end{array}\right]\\ \;\left(\left[\begin{array}{cc} 0 & 0\\ I & -B_{\theta_{k},k}^{\delta } \end{array}\right]\left[\begin{array}{c} x_{k+1}\\ u_{k} \end{array}\right]-\left[\begin{array}{c} -I\\ F_{\theta_{k},k}^{\delta } \end{array}\right]x_{k}\right), \end{array}$$

where $F_{\theta_{k},k}^{\delta }$ and $B_{\theta_{k},k}^{\delta }$ are defined in Equation (28); ${\text{Ψ}}_{\theta_{k},k+1}=\overset{s}{\underset{j=1}{\sum }}P_{j,k+1}p_{ij,k}$; *P*_θ_*k*_, *k*_ is a positive definite matrix; *Q*_θ_*k*_, *k*_ and *R*_θ_*k*_, *k*_ are semi-definite weighting matrices. The solution to the optimization problem expressed in Equations (41) and (42) that guarantees the optimal state-control sequence ${\left\{\left(x_{\mu ,k+1}^{*},u_{\mu ,k}^{*}\right)\right\}}_{k=0}^{N-1}$ for a fixed instant *k* and state θ_*k*_, is given by the following Robust Regulator for Discrete Markov Jump Linear Systems (RR-DMJLS):

**Robust Regulator for DMJLS (Cerri and Terra**, **2017****)**.
**Initial Conditions:** Set *x*_0_, θ_0_, ℙ, $P_{i}\left(N\right)≻0$, ∀*i* ∈ Θ: = {1, …, *s*}.
**Step 1:** (Backward). Calculate for all k=N-1,…,0:
  (43) $$\begin{array}{l} \Psi_{i,k+1}=\sum_{j=1}^{s}\;P_{j,k+1}\;p_{ij,k}\\ \left[\begin{array}{c} L_{\mu ,i,k}\\ K_{\mu ,i,k}\\ P_{\mu ,i,k} \end{array}\right]={\left[\begin{array}{ccc} 0 & 0 & 0\\ 0 & 0 & 0\\ 0 & 0 & -I\\ 0 & 0 & {\hat{F}}_{i,k}\\ I & 0 & 0\\ 0 & I & 0 \end{array}\right]}^{T}{\left[\begin{array}{cccccc} \Psi_{i,k+1}^{-1} & 0 & 0 & 0 & I & 0\\ 0 & R_{i,k}^{-1} & 0 & 0 & 0 & I\\ 0 & 0 & Q_{i,k}^{-1} & 0 & 0 & 0\\ 0 & 0 & 0 & W_{i,k} & \hat{I} & -{\hat{B}}_{i,k}\\ I & 0 & 0 & {\hat{I}}^{T} & 0 & 0\\ 0 & I & 0 & -{\hat{B}}_{i,k}^{T} & 0 & 0 \end{array}\right]}^{-1}\\ \;\left[\begin{array}{c} 0\\ 0\\ -I\\ {\hat{F}}_{i,k}\\ 0\\ 0 \end{array}\right], \end{array}$$
**Step 2:** (Forward). Obtain for each k=0,…N-1:
  $\left[\begin{array}{c} x_{k+1}^{*}\\ u_{k}^{*} \end{array}\right]=\left[\begin{array}{c} L_{\mu ,i,k}\\ K_{\mu ,i,k} \end{array}\right]x_{k}^{*}$.

Equation (43) uses the following auxiliary matrices:

(44) $$\begin{array}{l} \begin{array}{c} W_{i,k} \end{array}=\left[\begin{array}{cc} \mu^{-1}I-{\hat{\lambda }}_{i,k}^{-1}H_{\theta_{k},k}H_{\theta_{k},k}^{T} & 0\\ 0 & {\hat{\lambda }}_{i,k}^{-1}I \end{array}\right],\\ \hat{I}=\left[\begin{array}{c} I\\ 0 \end{array}\right],\;{\hat{B}}_{i,k}=\left[\begin{array}{c} B_{\theta_{k},k}\\ E_{B_{\theta_{k},k}} \end{array}\right],\;{\hat{F}}_{i,k}=\left[\begin{array}{c} F_{\theta_{k},k}\\ E_{F_{\theta_{k},k}} \end{array}\right],\\ \lambda_{i,k}>∥\mu H_{\theta_{k},k}^{T}H_{\theta_{k},k}∥. \end{array}$$

In this formulation, μ > 0 is a penalty parameter responsible to guarantee the robustness of the RR-DMJLS. In fact, when μ → +∞ then $W_{i,k}\to 0$. In consequence, the DMJLS closed-loop response is given by:

(45) $$\{\begin{array}{c} L_{\infty ,\theta_{k},k}=F_{\theta_{k},k}+B_{\theta_{k},k}K_{\infty ,\theta_{k},k}\\ E_{F_{\theta_{k},k}}+E_{B_{\theta_{k},k}}K_{\infty ,\theta_{k},k}=0, \end{array}$$

which provides the robust optimal response $\left(x_{k+1}^{*},u_{k}^{*}\right)$. Details of the necessary and sufficient conditions for existence of the mean square stabilizing solution and robustness of this regulator can be found in Cerri and Terra (2017).

Let μ = 9.998 × 10^6^ and $\lambda_{i,k}=1\times 10^{17}$ in order to satisfy Equations (44) and (45); weighting matrices *R*_1, *k*_ = *R*_2,*k*_ = *R*_3,*k*_ = 1, *Q*_1,*k*_ = *Q*_2,*k*_ = *Q*_3,*k*_ = *I*_5_ and $P_{1}\left(N\right)=P_{2}\left(N\right)=P_{3}\left(N\right)=1\times 10^{10}\times I_{5}$; and the probability matrix ℙ defined in Equation (36). By using the robust regulator presented in Equation (43), we obtain the following control law:

(46) $$u_{k}=K_{\infty ,\theta_{k},k}\;x_{k}=K_{a,\theta_{k}}x_{a_{k}}+K_{int,\theta_{k}}x_{int_{k}},$$

where *K*_*a*,θ_*k*__ is the gain to the states *x*_*a*_*k*__ and *K*_*int*,θ_*k*__ is the gain to the state *x*_*int*_*k*__. Table 2 shows the control gains obtained for three Markovian modes. We do not consider uncertainties in the third term of *E*_*F*_*i,k*__, as a consequence, the algorithm decouples the state variable ϕ_*m*_ guaranteeing the controllability of the system (see *K*_3_).

**Table 2:** Control gains.

**Markov modes**	***K*_1_**	***K*_2_**	***K*_3_**	***K*_4_**	***K*_*int*_**	**Gains**
θ = 1	[−0.0073	−2.787	0	0	11.027 ]	= [*K*_*a*, 1_*K*_*int*_]
θ = 2	[−0.0073	−11.149	0	1.213	74.983 ]	= [*K*_*a*, 2_*K*_*int*_]
θ = 3	[−0.0065	−16.724	0	1.516	79.394 ]	= [*K*_*a*, 3_*K*_*int*_]

Considering the control law presented in Equation (46), the closed-loop response for Equation (28) without considering the disturbance τ_*h*_*k*__, is given by:

(47) $$\begin{array}{l} \left[\begin{array}{c} x_{a_{k+1}}\\ x_{int_{k+1}} \end{array}\right]=\underset{F_{\theta ,k}+B_{\theta ,k}K_{\theta_{k}}}{\underset{︸}{\left[\begin{array}{cc} F_{a_{\theta ,k}}+B_{a_{\theta ,k}}K_{a,\theta_{k}} & B_{a_{\theta ,k}}K_{int,\theta_{k}}\\ C_{a}T_{s} & 1 \end{array}\right]}}\left[\begin{array}{c} x_{a_{k}}\\ x_{int_{k}} \end{array}\right]+B_{r_{\theta ,k}}r_{k}+\\ \;\underset{\delta F_{\theta ,k}+\delta B_{\theta ,k}K_{\theta_{k}}=H_{k}\Delta_{k}(E_{F_{k}}+E_{B_{k}}K_{\infty ,\theta_{k},k})=0}{\underset{︸}{\left[\begin{array}{cc} \delta F_{11_{\theta ,k}}+\delta B_{11_{\theta ,k}}K_{a,\theta_{k}} & \delta F_{12_{\theta ,k}}+\delta B_{11_{\theta ,k}}K_{int,\theta_{k}}\\ \delta F_{21_{\theta ,k}}+\delta B_{21_{\theta ,k}}K_{a,\theta_{k}} & \delta F_{22_{\theta ,k}}+\delta B_{21_{\theta ,k}}K_{int,\theta_{k}} \end{array}\right]}}\left[\begin{array}{c} x_{a_{k}}\\ x_{int_{k}} \end{array}\right], \end{array}$$

where δ*F*_*k*_ + δ*B*_*k*_*K*_∞,_θ_*k*__,*k*_ = 0 guarantees the robustness of the system according to Equation (45).

### 3.2. $H_{\infty }$ force control design

Consider the nominal model:

(48) $$G_{n}(s)=\frac{\rho N_{p}K_{s}K_{t}K_{PI}}{K_{PI}M_{meq}s^{2}+K_{PI}B_{meq}s+K_{s}}.$$

We use a mixed-sensitivity shaping approach $S={\left(1+G_{n}K_{c}\right)}^{-1}$ to ensure tracking performance and disturbance rejection, and *K*_*c*_*S* to limit the control signal (Skogestad and Postlethwaite, 2007). The $H_{\infty }$ problem is defined as:

(49) $$\begin{array}{l} \underset{K_{c}}{\min}\|\mathbb{N}(K_{c}){\|}_{\infty },\;\mathbb{N}={\left[\begin{array}{cc} w_{e}S & w_{u}K_{c}S \end{array}\right]}^{T}={\left[\begin{array}{cc} z_{1} & z_{2} \end{array}\right]}^{T},\\ \;\|\mathbb{N}{\|}_{\infty }=\max_{\omega }\bar{\sigma }(\mathbb{N}(j\omega ))\leq \gamma , \end{array}$$

where *w*_*e*_(*s*) and *w*_*u*_(*s*) are respectively performance and control weights, *K*_*c*_ is a stabilizing controller that bounds ℕ by an attenuation level γ, and $\bar{\sigma }\left(\mathbb{N}\right)$ is given by the usual Euclidean vector norm:

(50) $$\bar{\sigma }(\mathbb{N})=\sqrt{|w_{e}S{|}^{2}+|w_{u}K_{c}S{|}^{2}}.$$

We define the performance weighting *w*_*e*_(*s*) by:

(51) $$w_{e}=\frac{\text{s}/10^{\left(Ms/20\right)}+\omega_{b}}{\text{s}+\omega_{b}\epsilon_{s}},$$

where $\epsilon_{s}=10^{-5}$ is the maximum steady-state error, *M*_*s*_ = 2 dB is the maximum peak of *S*, and ω_*b*_ = 10π is related to the close-loop bandwidth. We determine the control input weighting *w*_*u*_(*s*) with

(52) $$w_{u}=1/M_{u},$$

where *M*_*u*_ = 523.6 rad/s (5,000 rpm) is the maximum value for *u*. The control system is considered according to Figure 4B. Based on Equations (51) and (52), the $H_{\infty }$ force controller *K*_*c*_(*s*) is obtained:

(53) $$K_{c}(s)=\frac{2.78\text{e}9s^{3}+2.18\text{e}12s^{2}+6.95\text{e}13s+6.95\text{e}10}{s^{4}+5.52\text{e}6s^{3}+3.34\text{e}11s^{2}+1.35\text{e}13s+4.3\text{e}6}.$$

Finally, the controller is discretized by using a zero-order hold, with *T*_*s*_ = 1 ms,

(54) $$K_{c}(z)=\frac{6.16z^{2}-12.11z+5.95}{z^{3}-1.96z^{2}+0.96z}.$$

## 4. Experimental results

In order to evaluate the stability and performance of the proposed control approaches, time and frequency response tests are performed with a healthy user for three different operation modes presented in Section 2.3. We show a comparative study of force controllers and impedance controllers, according to Figure 4. This study was approved and carried out in accordance with the recommendations of the Ethics Committee of the Federal University of São Carlos (Number 26054813.1.0000.5504).

### 4.1. Force controllers

In this section, robust force controllers presented in Section 3, RR-DMJLS and $H_{\infty }$, are compared. Two performance criteria are used for this purpose. We consider the rise time *t*_*r*_ and a normalized mean error between spring and desired forces defined as

(55) $$e_{qc}=\frac{1}{N}\sum_{k=1}^{N}\left|\frac{F_{d}-F_{s}}{max\{F_{d}\}}\right|\cdot 100\%,$$

where *N* = 8/*T*_*s*_ is the number of samples.

**Figure 6:**
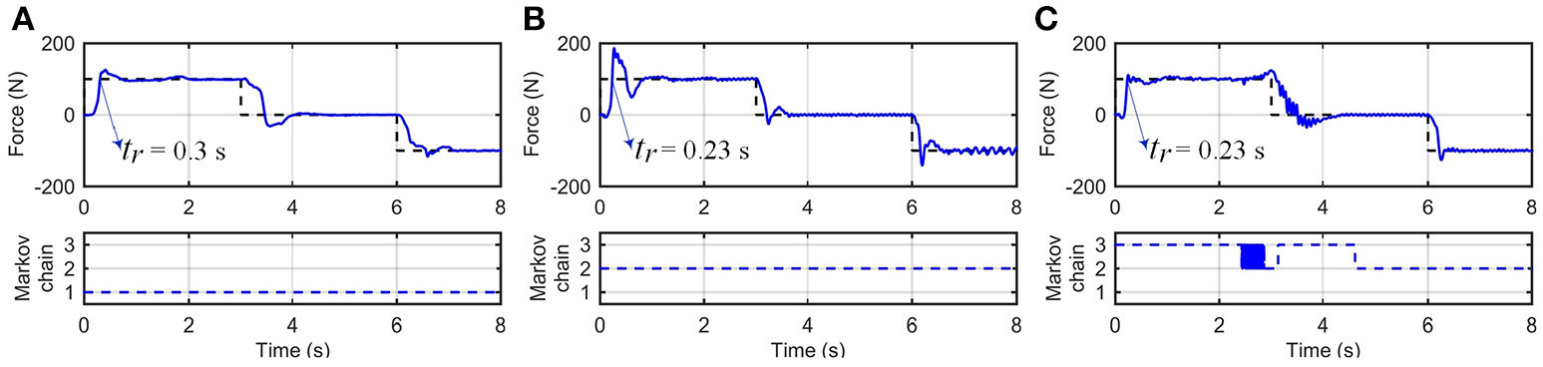
Recursive RR-DMJLS force controller. **(A)** Fixed mode. **(B)** Resistive mode. **(C)** Resistive and passive modes. For each operation mode, graphs show desired (black) and actual (blue) spring forces (top), and Markov chains (bottom).

Figure 6 shows time responses of the system using the RR-DMJLS force controller. In these experiments, three different levels (100, 0, and − 100 N) of desired forces, *F*_*d*_, are set during a total time of *T* = 8 s. In tests performed for fixed and resistive modes, shown in Figures 6A,B, the Markovian modes remained constant. Notice in the test shown in Figure 6C, the Markov chain changes between Modes 2 and 3. The RR-DMJLS responses are similar for three modes available, maintaining an appropriate tracking despite natural differences between human and robot dynamics. Notice that *t*_*r*_ are similar to the respective desired force levels and the mean errors *e*_*qc*_ are 14.24, 11.97, and 11.9%, for tests shown in Figures 6A–C, respectively.

**Figure 7:**
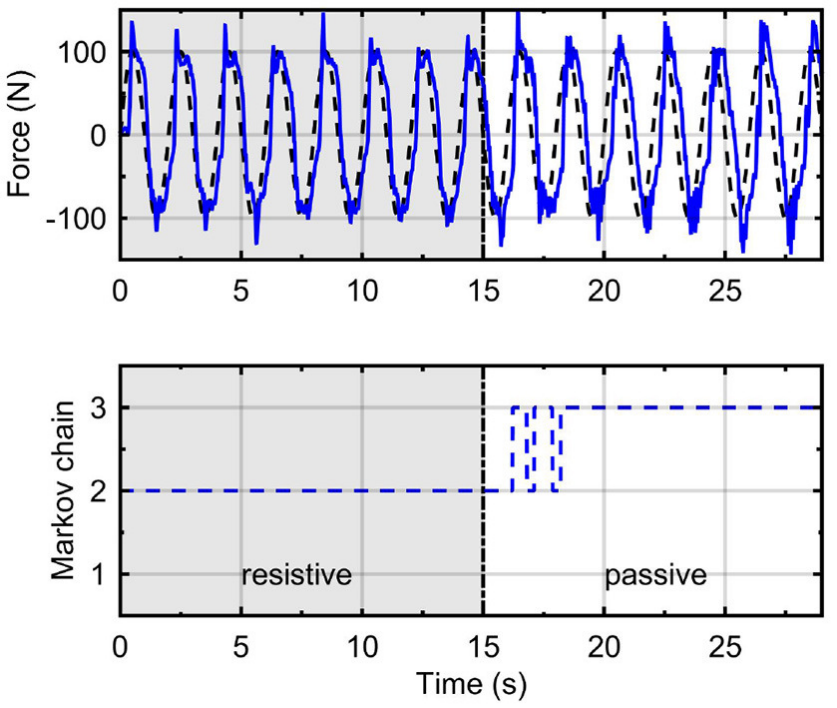
Recursive RR-DMJLS force controller. Force response (solid), sinusoidal force reference (dashed) with a change of operation mode (top). Markov chain (bottom).

Figure 7 shows the force control response of the RR-DMJLS while tracking a sinusoidal force reference. In this experiment, the user is asked to be resistive to the movement of the platform during the first fifteen seconds, and then to remain passive during the next fifteen seconds. We observe that force control maintains a similar response despite the abrupt change in the human dynamics (resistive → passive). Notice that jumps between Markovian states reflect time transitions between different operation modes of the system. However, for a condition where the operation modes change more frequently, these transitions would be detected with a certain delay. We hypothesize that this delay is related to the jump parameter identification method considered (Equation 37). It could be improved by estimating alternative variables. For example, the stiffness and damping parameters of the human being.

**Figure 8:**
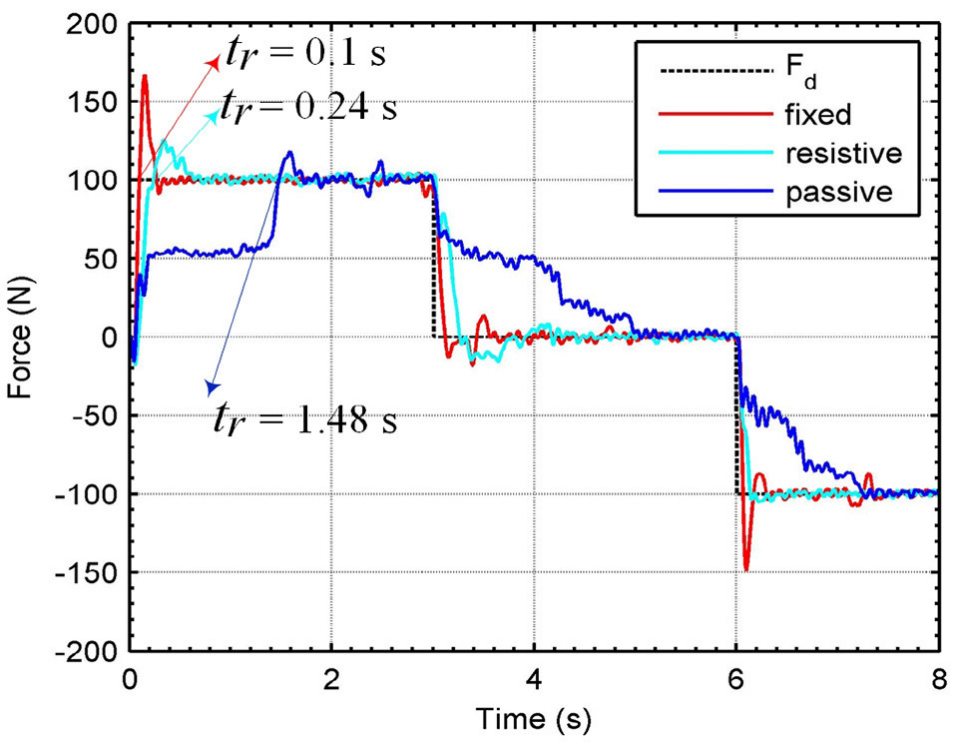
Time response of the system with $H_{\infty }$ force controller.

Figure 8 presents time responses of the system using the $H_{\infty }$ force control approach. In this case, also are applied three different levels of desired forces *F*_*d*_ in *T* = 8 s, for each operation mode: fixed, resistive, and passive. The rise time *t*_*r*_ is lower for fixed and resistive modes in comparison with the passive mode. The mean errors obtained for fixed, resistive and passive modes were 5.89, 8.23, and 23.29%, respectively. Comparing mean errors between both controllers, we obtain better performance for the $H_{\infty }$ force controller for the fixed mode. For the passive mode, the RR-DMJLS provides better performance. However, when we design the $H_{\infty }$ control based on the passive mode, it does not stabilize the system when it is operating in the fixed mode. An advantage of the RR-DMJLS is the uniformity of performance obtained for the whole system.

**Figure 9:**
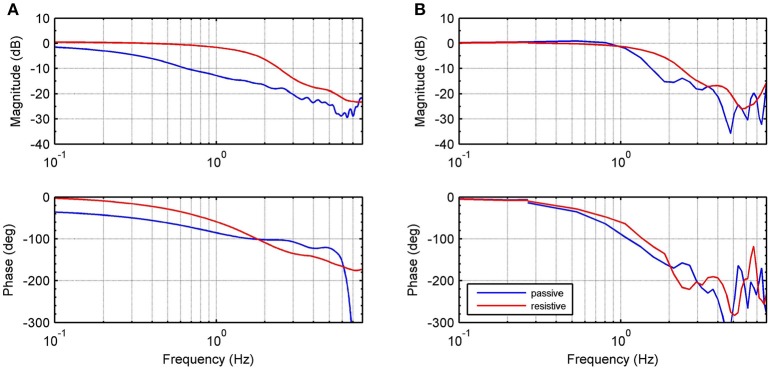
Frequency closed-loop response of the system with force control. **(A)** $H_{\infty }$ force control. **(B)** Recursive RR-DMJLS force control. Graphs show responses for passive (blue) and resistive (red) modes.

Figure 9 shows frequency responses of the closed-loop system using the $H_{\infty }$ and recursive RR-DMJLS force controllers. For passive and resistive modes, we apply a desired force signal given by a chirp signal with amplitude of 100 N, sweeping frequencies between 0 and 8 Hz. Some indexes for both controllers are compared. For the $H_{\infty }$ force controller, shown in Figure 9A, we have: maximum magnitude during passive mode, |*G*_*fp*_|_*max*_ = −1.6 dB, and resistive mode, |*G*_*fr*_|_*max*_ = 0.86 dB; bandwidth for passive mode, *B*_*w*_*fp*_−3*dB*___ = 0.2 Hz, and bandwidth for resistive mode, *B*_*w*_*fr*_−3*dB*___ = 1.39 Hz; phase at cut-off frequency for passive mode, $\alpha_{fp}=-43^{{}^{\circ}}$, and for resistive mode, $\alpha_{fr}=-79^{{}^{\circ}}$. For the frequency response of the RR-DMJLS shown in Figure 9B, we obtain: |_*G*_*fp*_|*max*_ = 0.9 dB; |_*G*_*fr*_|*max*_ = 0.32 dB; *B*_*w*_*fp*_−3*dB*___ = 1.2 Hz; *B*_*w*_*fr*_−3*dB*___ = 1.5 Hz; $\alpha_{fp}=-110^{{}^{\circ}}$; and $\alpha_{fr}=-75^{{}^{\circ}}$. Notice that the bandwidth of the closed-loop system is greater for the RR-DMJLS controller.

### 4.2. Impedance control

**Figure 10:**
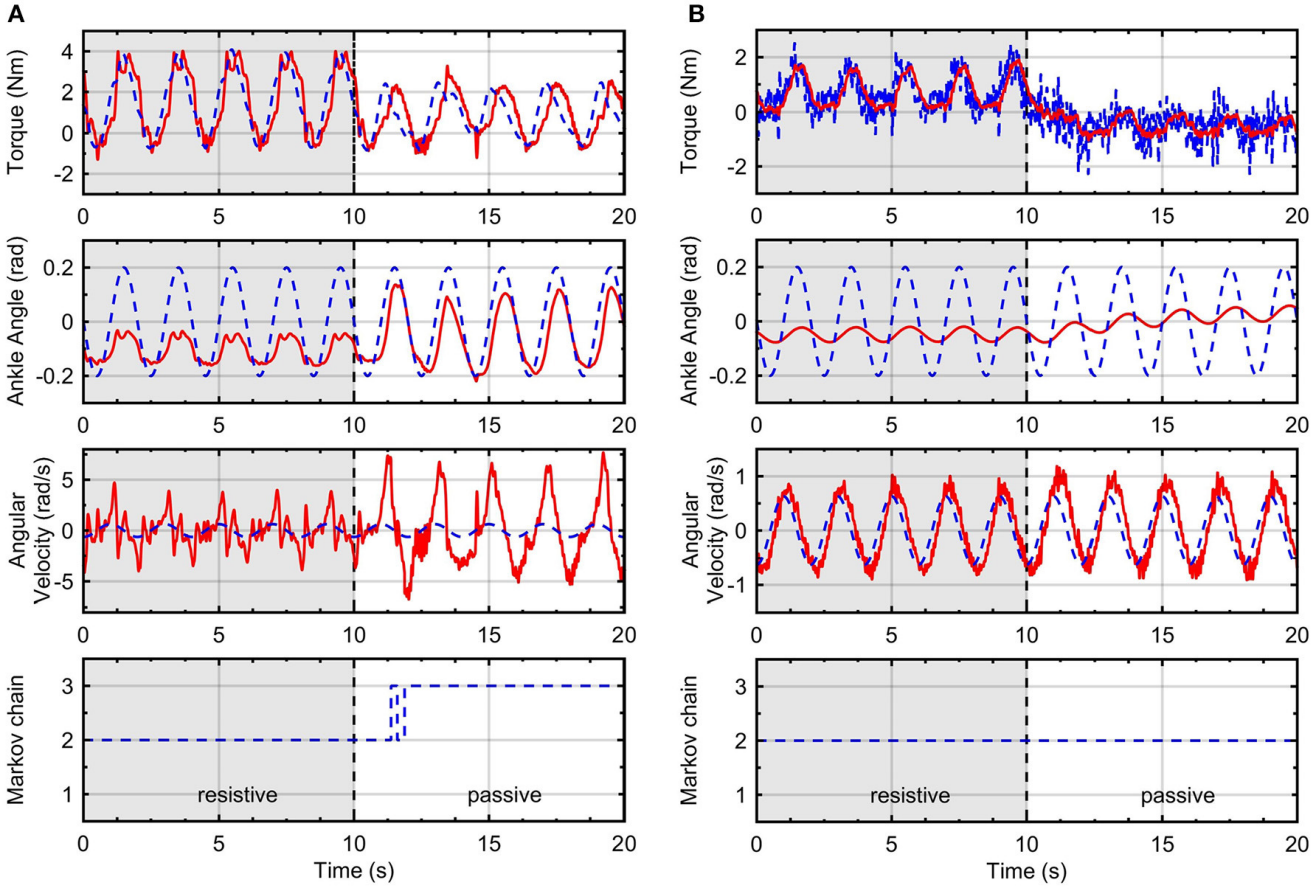
Impedance control with inner RR-DMJLS force control. Torque and impedance control responses for: **(A)** *K*_*v*_ = 15 N·m/rad and *B*_*v*_ = 0 N·m·s/rad, and **(B)** *K*_*v*_ = 15 N·m/rad and *B*_*v*_ = 5 N·m·s/rad. Graphs show desired (blue) and measured (red) values for the platform torque (top), angular position of the ankle joint (middle-top), angular velocity of the ankle joint (middle-bottom); and Markov chain (bottom).

To obtain the RR-DMJLS force control response during impedance controlled movements, we perform two experiments in which the system must track a kinematic reference. In both cases, the user was asked to be resistive during first ten seconds and then to be passive during the next ten seconds. Figure 10A shows the force and impedance control for a pure stiffness control configuration and Figure 10B a stiffness-damping control configuration. Notice that the torque tracking performance is similar for two modes in spite of the transition between them. Regarding the passive case, the position tracking is better for the pure-stiffness configuration. However, since this configuration has no damping parameter, the velocity tracking is worse. We can include a damping coefficient. However, it can decrease the performance of the position tracking. Thus, there exists a compromise between these control objectives that must be considered by the assistance strategy. Regarding the Markovian states, notice that increasing *B*_*v*_ it reduces the velocity of the system and enforces the system to remain in the same Markovian state, Figure 10B.

**Figure 11:**
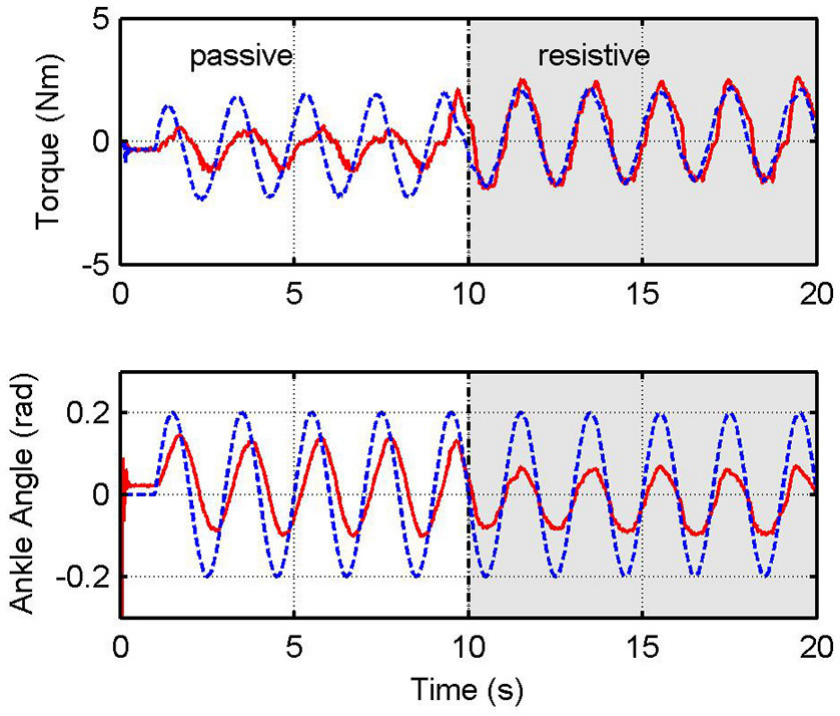
Impedance control with inner $H_{\infty }$ force control. Graphs show desired (blue) and measured (red) values for the platform torque, τ_*plat*_ (top), and angular position of the ankle joint (bottom).

Figure 11 shows time responses using the impedance control with the inner $H_{\infty }$ force control loop, for *K* = 15 N·m/rad, following a sinusoidal trajectory for ankle angular position. In this case, the user is asked to remain passive during the first ten seconds, and then to be resistive during the next ten seconds. Results show that the torque tracking performance is worse when the human being is in passive mode.

In order to quantify the performance achieved by the proposed controllers, we compute the real stiffness and damping parameters of the system. For this purpose, we calculate the torque τ_*K*_*v*__ generated by the virtual stiffness *K*_*v*_ and the torque τ_*B*_*v*__ generated by the virtual damping *B*_*v*_ with

(56) $$\tau_{K_{v}}=\tau_{plat}-B_{v}\omega_{e}\text{ and }\tau_{B_{v}}=\tau_{plat}-K_{v}\phi_{e},$$

where $\phi_{e}=\phi_{l}^{d}\;-\;\phi_{l}$ is the angular position error and $\omega_{e}=\omega_{l}^{d}\;-\;\omega_{l}$ is the angular velocity error. We compute the root mean square (RMS) errors of the measured variables in the experiments shown in Figures 10, 11. The RMS of the angular error ϕ_*e*_ is given by:

(57) $$\text{RMS}\{\phi_{e}\}=\sqrt{\frac{1}{N}\sum_{k=1}^{N}\phi_{e_{k}}^{2}},$$

where *N* = 10/*T*_*s*_ is the number of samples in each test (passive or resistive). In a similar way, we calculate RMS values for angular velocity error ω_*e*_, torque generated by virtual stiffness τ_*K*_*v*__, and damping τ_*B*_*v*__. Notice that, when *B*_*v*_ = 0 then τ_*B*_*v*__ = 0 and τ_*K*_*v*__ = τ_*plat*_. Actual stiffness *K*_*r*_ and damping *B*_*r*_ are given by:

(58) $$K_{r}=\frac{\text{RMS}\{\tau_{K_{v}}\}}{\text{RMS}\{\phi_{e}\}}\text{ and }B_{r}=\frac{\text{RMS}\{\tau_{B_{v}}\}}{\text{RMS}\{\omega_{e}\}},$$

see Table 3. The error between the virtual stiffness and the actual stiffness are calculated, *e*_*K*_*v*__ = |(*K*_*v*_ − *K*_*r*_)/*K*_*v*_|100% and the error between the virtual damping and the actual damping, *e*_*B*_*v*__ = |(*B*_*v*_ − *B*_*r*_)/*B*_*v*_|100%. We compare results of the impedance control with *K*_*v*_ = 15 Nm/rad and *B*_*v*_ = 0 Nms/rad. Impedance control with RR-DMJLS presents higher stiffness accuracy in both passive and resistive modes, 15.28 Nm/rad and 16.13 Nm/rad, respectively. With $H_{\infty }$ controller, it provides 16.44 Nm/rad in passive mode and 7 Nm/rad in resistive mode. Stiffness errors *e*_*K*_*v*__ of the RR-DMJLS force control are smaller than the $H_{\infty }$ force control. This difference can be seen in the passive mode (θ = 3), where the *e*_*K*_*v*__ error of the $H_{\infty }$ is approximately seven times greater than the RR-DMJLS. In the test with *K*_*v*_ = 15N·m/ rad and *B*_*v*_ = 5N·m·s/ rad, the performance is preserved.

**Table 3:** Experimental measurements of the system stiffness and damping.

**Test with inner $H_{\infty }$**force control**,** ***K***_*****v*****_ **= 15 and** **B**_*****v*****_ **= 0**.			**Test with inner** ***RR*****-** ***DMJLS*** **force control**, ***K***_*****v*****_ **= 15 and** ***B***_*****v*****_ **= 0**.	
	*K*_*r*_	*e*_*K*_*v*__	*K*_*r*_	*e*_*K*_*v*__
θ	$\left(\frac{N\cdot m}{rad}\right)$	(%)	$\left(\frac{N\cdot m}{rad}\right)$	(%)
2	16.44	9.60	15.28	1.86
3	7.00	53.33	16.13	7.53
**Test with inner** ***RR*****-*****DMJLS*** **force control**, ***K***_***v***_ **= 15 and** ***B***_***v***_ **= 5**.				
	*K*_*r*_	*e*_*K*_*v*__	*B*_*r*_	*e*_*B*_*v*__
θ	$\left(\frac{N\cdot m}{rad}\right)$	(%)	$\left(\frac{N\cdot m\cdot s}{rad}\right)$	(%)
2	15.38	2.53	4.86	2.80
3	15.91	6.06	4.70	6.00

**Figure 12:**
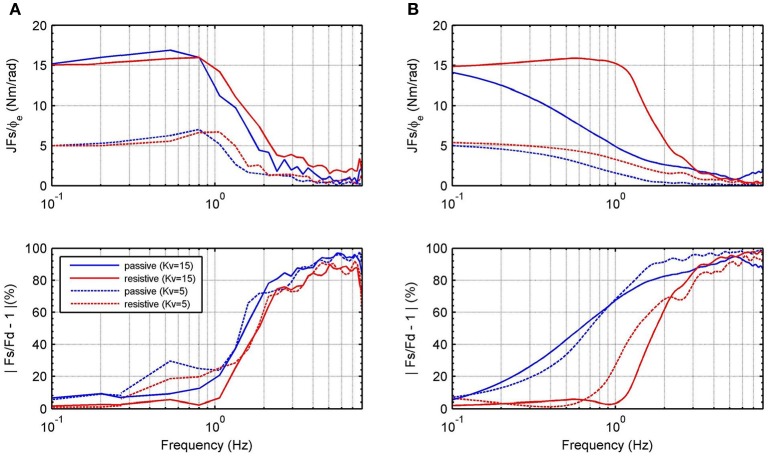
Frequency response for the measured impedance, $JF_{s}/\phi_{e}$, and force error, |*F*_*s*_/*F*_*d*_ − 1| · 100%, for *K*_*v*_ = 15, *K*_*v*_ = 5 and *B*_*v*_ = 0. **(A)** Inner recursive RR-DMJLS force control. **(B)** Inner $H_{\infty }$ force control.

Figure 12 shows the frequency response for the impedance which is measured between output torque and angular position error. For passive and resistive modes, we apply a desired angular position trajectory given by a chirp signal with amplitude 0.2 rad, sweeping frequencies between 0 and 8 Hz. In these experiments, impedance controller is defined as pure stiffness configuration; therefore, magnitude of the impedance should be almost constant. Notice that it is guaranteed only until 1 Hz. Figure 12A shows the behavior of the RR-DMJLS-based impedance control. Figure 12B shows the frequency response for the $H_{\infty }$-based impedance control. Notice for this controller that the performance decreases when the system operates in the passive mode. We can see that the RR-DMJLS is a more resilient impedance control if compared with $H_{\infty }$-based control for different operating modes and desired impedances.

## 5. Discussion and conclusions

The model-based robust force control approach was evaluated in a robotic platform for ankle rehabilitation, improving its impedance control performance. First, the robot-human dynamics was modeled considering the ankle impedance a second-order system with inertia, stiffness, and damping parameters. Since we do not have exact knowledge of the human parameters, our system is subject to parametric uncertainties and we have defined three operation modes related to the human-robot activity whose time transitions were modeled via a Markov chain. For comparison purposes, an $H_{\infty }$ force controller was also evaluated. Although it can guarantee coupled stability, the force control performance decreases when the system is in the passive operation mode. We have also designed and implemented an RR-DMJLS for dealing with abrupt changes and system uncertainties by guaranteeing robust mean square stability of the system. Experimental results show RR-DMJLS outperformed the $H_{\infty }$ force controller.

### 5.1. Related work

The impedance control configuration used is based on Hogan (1985) and it is aimed at the regulation of the dynamical behavior in the interaction port by variables that do not depend on the environment. The actuator together with the controller are modeled as an impedance, *Z*_*r*_, with velocity inputs (angular) and force outputs (torque). The environment is considered an admittance, *Y*_*e*_, in the interaction port. Colgate and Hogan (1988, 1989) presented sufficient conditions for the determination of stability of two coupled systems and explained how two physically coupled systems with *Z*_*r*_ and *Y*_*e*_ with passive port functions can guarantee stability. These concepts have been useful for the implementation of interaction controls for almost three decades. The stability of two coupled systems is given by *Z*_*r*_ and *Y*_*e*_ eigenvalues and the performance is evaluated by through the impedance *Z*_*r*_.

Buerger and Hogan (2007) described a methodology in which an interaction control is designed for a robot module used for rehabilitation purposes. They considered an environment with restricted uncertain characteristics, therefore the admittance is rewritten as *Y*_*e*_(*s*) = *Y*_*n*_(*s*) + *W*(*s*)Δ(*s*). The authors also used a second-order dynamics to model the stiffness, damping, and inertia of the human parameters. Complementary stability for interacting systems was defined, where stability is determined by an environment subject to uncertainties. Therefore, a coupled stability problem is considered a robust stability problem.

Regarding the human modeling, the dynamic properties of the lower limbs and muscular activities vary considerably among subjects. This is relevant since SRPAR has been designed for users that suffer diseases that affect the human motor control system, e.g., stroke and other conditions that cause hemiplegia. Typically, such diseases change stiffness and damping in the ankle and knee joints, hence producing spasticity or hypertonia (Lin et al., 2006; Chow et al., 2012). Therefore, the development of a control strategy that guarantees a safe interaction between patient and platform, mainly in virtue of uncertainties related to the human being, is fundamental.

Li et al. (2017) and Pan et al. (2017) proposed adaptive control schemes for SEA-driven robots. They considered two operation modes in the adaptation process, namely robot-in-charge and human-in-charge, which are close related to the passive and resistive operation modes, respectively, proposed in this paper. However, the control adaptation is based on changes in the desired position input of the SEA controller and estimation of coordinate accelerations through nonlinear filtering.

In human-robot interaction control systems, the efficiency of the force actuator operation deserves special attention. Although SEAs are characterized by a low output impedance, an important requirement for improving such efficiency is the achievement of a precise and proportional output torque with respect to the desired input. Pratt (2002), Au et al. (2006), Kong et al. (2009), Mehling and O'Malley (2014), and dos Santos et al. (2015) developed force controllers for ankle actuators using SEA. In this paper, we proposed a force control methodology that can deal with system uncertainties and guarantee robust mean square stability. Similar performance was obtained in different tests performed. Accuracies of 98.14% for resistive mode and 92.47% for passive mode were obtained in the pure stiffness configuration. In the stiffness-damping configuration, with *K*_*v*_ = 15 and *B*_*v*_ = 5, the accuracy obtained in the resistive case was of 97.47% for stiffness and 97.2% for damping, and in the passive case was of 93.94% for stiffness and 94% for damping.

On the contrary, using a fixed-gain control approach based on $H_{\infty }$ synthesis, the performance was not similar among operation modes. We showed that this strategy can guarantee coupled stability; nevertheless, force control performance decreases when the system is in the passive operation mode. This is reflected in the impedance control accuracy for the pure stiffness configuration, falling from 90.4% in the resistive mode to 46.67% in the passive mode.

### 5.2. Shortcomings and possible improvements

In order to control the SRPAR, we proposed a methodology to force control based on RR-DMJLS. It considers a discrete-time Markovian model with three states associated with the operating modes of the system. The model was augmented for eliminating the steady state error through the inclusion of an integral action. We also found appropriate uncertain matrices considering frequency responses for relative errors between perturbed and nominal models.

Regarding the observation of the operation modes, in Figure 10A there exists a delay in the jump identification from resistive to passive mode, and in Figure 10B the proposed jump identification method was not even able to identify the jump between modes. This behavior is directly related to virtual damping *B*_*v*_ selected since load angular velocity $w_{l_{k}}^{f}$ is lower than 2 rad/s. A possible solution to the problem is the estimation of the virtual stiffness and damping of the human being for the definition of the bounds of the identification method.

Based on our observations of the system behavior, we have defined a probability matrix ℙ that models those transitions among different modes. We hypothesize the probabilities may vary in function of the user's physiological conditions, therefore, our probability matrix can be considered partially or completely uncertain. In a future study, we aim at using our methodology subject to uncertain transition probabilities with the unknown Markov chain proposed in Bortolin and Terra (2016).

Other approaches may improve our methodology. For example, a disturbance observer, as proposed in Kong et al. (2009), could compensate the effect of human torque τ_*h*_ in Equations (14) and (20). Optimal robust filter for DMJLS (Ishihara et al., 2015) and extended robust Kalman filter proposed in Inoue et al. (2016) could better estimate the states of the SRPAR.

## Author contributions

AJ, JJ, FE, and JP conceived research, performed the experiments and the data analysis, drafted the manuscript, and coded the controllers. MT and AS participated in the design of the controllers and in the draft of the manuscript. All authors read and approved the final manuscript.

### Conflict of interest statement

The authors declare that the research was conducted in the absence of any commercial or financial relationships that could be construed as a potential conflict of interest.
